# Prevalence of Anxiety and Depression in Pakistani Patients With Acne Vulgaris

**DOI:** 10.7759/cureus.78734

**Published:** 2025-02-08

**Authors:** Ayesha Jamil, Mariam Sheikh, Mahwash Rana, Sabeen Aftab, Muhammad Atif Qureshi, Osama Habib, Nauman Ismat Butt

**Affiliations:** 1 Dermatology, Azra Naheed Medical College, Lahore, PAK; 2 Dermatology, Akhtar Saeed Medical and Dental College, Lahore, PAK; 3 Dermatology, Continental Medical College, Lahore, PAK; 4 Internal Medicine, Azra Naheed Medical College, Lahore, PAK; 5 Internal Medicine and Medical Education, Azra Naheed Medical College, Lahore, PAK; 6 Psychiatry, Azra Naheed Medical College, Lahore, PAK; 7 Internal Medicine/Rheumatology, Azra Naheed Medical College, Lahore, PAK

**Keywords:** acne vulgaris, anxiety, depression, gags, socio-demographic factors

## Abstract

Introduction

Adolescents with acne vulgaris, a prevalent dermatological condition, have a significant prevalence of anxiety and depression, which can have negative effects on their quality of life.

Objectives

The purpose of this study was to find out the prevalence of anxiety and depression in Pakistani patients with acne vulgaris.

Methods

This six-month cohort study was conducted at Chaudhry Muhammad Akram Teaching and Research Hospital in Lahore, Pakistan, involving 250 acne vulgaris patients (aged 16-40) and 200 age- and sex-matched controls. Exclusion criteria included certain medical and psychological conditions. The Hospital Anxiety and Depression Scale (HADS) and socio-demographic questionnaires were utilized for data collection. The severity of the acne was evaluated with the help of the Global Acne Grading System (GAGS). The final data obtained were analyzed with IBM SPSS Statistics for Windows, Version 27.0 (Released 2020; IBM Corp., Armonk, New York, United States), employing the Kruskal-Wallis test, chi-squared test, and independent t-test.

Results

Among 450 participants, 302 (67.1%) were female, with a majority aged 23-33 years old. A significant difference in marital status was found, with more acne cases being single (174, 69.6%) compared to controls (107, 53.5%; p<0.001). Most acne cases (154, 61.6%) had moderate severity. The mean anxiety HADS score was higher in the case group (17.62±4.85) compared to controls (6.74±4.40; p=0.027), while depression HADS scores did not significantly differ (p=0.643). Acne severity did not significantly correlate with anxiety or depression HADS scores.

Conclusion

Increased anxiety in individuals with acne vulgaris, particularly among females, was seen, while depression levels were similar between cases and controls.

## Introduction

Acne vulgaris is among the chronic skin disorders manifested by inflammation of the pilosebaceous unit, resulting in the development of comedones, erythematous papules, and cysts, with or without the presence of scar [1]. With a prevalence of up to 85% in teenagers, it is common among young adults and adolescents between the ages of 15 and 25. Although it can also appear on the back, shoulders, and upper chest, it primarily manifests on the face [2]. Acne can be classified in several ways: by the type of lesion (comedonal acne, which includes blackheads and whiteheads; inflammatory acne, including papules and pustules; and nodulocystic acne, which involves larger, painful cysts and nodules); by severity (mild acne with minimal lesions, moderate acne with a mix of lesions, and severe acne with extensive cysts or nodules that may lead to scarring); by underlying causes (hormonal acne, acne vulgaris due to excess oil and bacteria, acne mechanica triggered by friction or sweating, and occupational or cosmetic acne caused by chemicals or products); and by rare forms like acne conglobata and acne fulminans, which are more severe and require specialized treatment. Furthermore, there are two varieties of post-adolescent acne: late-onset papules, which appear after the 25-year age group, and persistent acne, which begins in adolescence and lasts into adulthood [3]. Because acne often appears on the face and affects the self-perception of the body and facial appearance, it imposes a significant psychological burden [4]. This may be due to the fact that the face, which draws visual attention, is a powerful tool for social communication [3].

Acne patients frequently experience feelings of embarrassment, low confidence, sadness, stress, and anxiety, especially after receiving multiple treatments, because they may become frustrated and disheartened by the lack of significant improvement, leading to concerns about their appearance and the emotional toll of feeling like their efforts are not yielding results. They also report embarrassment over recurring lesions, particularly redness and scarring [3]. Acne vulgaris ranks as the eighth most common condition globally, with an incidence rate of 9.4%, according to the Global Burden of Disease report [5]. Up to 56% of adolescents have acne vulgaris [6]. This condition adversely impacts daily functioning, interpersonal relationships, and self-assessment. Patients with acne vulgaris report psychological issues such as stress (50%), anxiety (44%), depression (18%), suicidal thoughts (6%), body dysmorphic disorder (8%), hostility, low self-esteem, and lasting personality impacts [7].

Studies indicate that up to 40% of acne patients experience anxiety and depression, with 6-7% reporting suicidal thoughts [8]. The increased psychiatric comorbidities seen in chronic conditions, such as depression, anxiety, and stress, contribute to this trend by amplifying feelings of frustration and emotional distress, further impacting a patient's mental well-being and exacerbating the negative effects of acne [9]. A review study analyzed 42 research and concluded that individuals with acne had a higher occurrence rate of anxiety level and depression compared to controls (p<0.0001), with a strong correlation between adult acne and these conditions [10]. In a study conducted in India, Bondade et al. found that 40% of acne patients had psychiatric morbidity, with 34% experiencing depressive symptoms and 6% anxiety [8]. Another study on psychiatric morbidity in acne patients reported clinically significant depression in 39.1% of cases and anxiety in 4.35% [11].

Psychological burdens, including anxiety, depression, and low self-esteem, are closely associated with acne [12]. Compared to patients with other dermatological conditions, those with acne have higher rates of anxiety and depression, with 30.2% affected [13]. The lifelong psychological impacts of acne can include low self-esteem, distorted self-confidence, dissatisfaction with personality, and difficulty in social interactions [14]. Thus, effective management of acne, which includes assessing psychological well-being, may help boost self-esteem and confidence [15].

The Aga Khan University Anxiety and Depression Scale (AKUADS) was employed in the current research to assess patients' anxiety and depression levels. Its use in this study is supported by its cultural sensitivity and reliability in detecting psychological symptoms in Pakistani patients [16]. The Hospital Anxiety and Depression Scale (HADS) is a self-assessment scale that has been developed and found to be a reliable instrument for detecting states of depression and anxiety in a hospital medical outpatient clinic setting [17]. The purpose of this study is to evaluate the anxiety and depression levels in individuals with acne vulgaris and examine the correlation between these psychological conditions and acne severity. 

This study contributes to the existing literature by specifically exploring the correlation between anxiety and depression levels and acne severity in individuals with acne vulgaris within the Pakistani population, a demographic that has not been extensively studied in this context. While several studies have suggested a link between acne and psychological distress, many have focused primarily on depression or anxiety in isolation, without analyzing the full spectrum of psychological comorbidities in relation to varying degrees of acne severity. This study aims to fill that gap by evaluating both anxiety and depression levels in individuals with acne, providing a more comprehensive understanding of how psychological conditions are intertwined with acne severity and whether more severe cases are associated with heightened mental health challenges in this particular population. This study hypothesizes that higher acne severity will be significantly correlated with higher levels of anxiety and depression in the Pakistani population, suggesting that individuals with more severe acne vulgaris will experience greater psychological distress compared to those with milder forms of acne.

## Materials and methods

This prospective cohort study was conducted in the Department of Dermatology at Chaudhry Muhammad Akram Teaching and Research Hospital in Lahore, Pakistan, from February 1 to July 31, 2024. A total of 250 patients diagnosed with acne vulgaris, aged 16-40, were included. The age group of 16-40 years was selected because acne vulgaris typically begins in adolescence, around age 16, due to hormonal changes and can persist into young adulthood, making this range ideal for assessing both the onset and long-term psychological impact of acne across key life stages. 

The exclusion criteria were implemented to ensure the accuracy and validity of the study results. Mental retardation was excluded to ensure participants could accurately complete the psychological assessments of anxiety and depression. Pregnancy and lactation were excluded due to hormonal fluctuations that significantly impact both acne severity and psychological well-being, which could confound the study's findings. Participants with other dermatological conditions like eczema, psoriasis, or rosacea were excluded, as these conditions can present with similar skin symptoms but have different causes and treatments, potentially affecting the relationship between acne vulgaris and psychological factors. Individuals with chronic medical conditions such as diabetes, thyroid disorders, or polycystic ovary syndrome (PCOS) were excluded as these conditions can influence both skin health and mental health. A history of mental health conditions was another exclusion criterion, as pre-existing psychological disorders could influence the results, making it difficult to isolate the impact of acne on mental well-being. Patients using medications that aggravate acne, like steroids or anticonvulsants, were excluded to prevent these drugs' confounding effects on the severity of acne and psychological distress. Lastly, acne due to steroid use was excluded due to its distinct hormonal influence, which differs from the mechanisms behind acne vulgaris, ensuring that the study focused solely on the psychological impact of acne vulgaris.

Additionally, 200 age- and gender-matched controls were recruited. The control group was selected from hospital staff and patients' relatives who shared similar socio-demographic characteristics (such as age, gender, and social background) to the acne vulgaris group. These participants were carefully screened to ensure they had no history of acne vulgaris or any other dermatological conditions that could affect the skin or mental health. Additionally, controls were free from chronic medical conditions or psychiatric disorders that could influence anxiety or depression levels, ensuring that the comparison with the acne group reflected the psychological impact of acne itself, without the influence of other health issues.

Using the Global Acne Grading System (GAGS), created by Doshi et al. in 1997 [18], a dermatologist evaluated the individuals' acne severity. Using six anatomical areas, forehead, chin, nose, left cheek, right cheek, and chest and upper back, the GAGS assesses primary acne lesions based on their location and clinical characteristics. According to surface area and distribution, each anatomical region, forehead, right cheek, left cheek, nose, chin, and chest and upper back, is given a factor of 2, 2, 2, 1, 1, and 3, respectively. With comedones worth 1, papules worth 2, pustules worth 3, and nodules worth 4, each acne lesion is assigned a score between 0 and 4. Lesion-free areas of the body receive a score of 0. Acne severity is divided into five categories by the total GAGS score: mild (1-18), moderate (19-30), severe (31-38), minimal (≥39), and no acne (0 points) [18].

The AKUADS was used for the screening of anxiety and depression. Both patients and matched controls completed the AKUADS. It is a culturally validated tool designed to assess both anxiety and depression in South Asian populations, specifically tailored for use in Pakistan. The AKUADS is available in Urdu, a language widely spoken in Pakistan, and was developed based on symptoms reported by patients with anxiety and depression at the Community Health Center (CHC) of Aga Khan University (AKU) [16]. It includes 25 items that cover psychological and somatic symptoms related to these conditions, helping to differentiate between anxiety and depression by categorizing items based on the type of symptoms they measure. The 25-item scale includes 12 mental and 13 physical symptoms and assesses the presence and severity of anxiety and depression over the past two weeks. The items are typically scored using a Likert-type scale ranging from 0 to 3, allowing participants to rate the frequency of their symptoms [16]. The total score is then used to assess the severity of the conditions, with higher scores indicating more severe symptoms of either anxiety or depression. While specific cutoff scores for diagnosis are not universally published, the scale usually classifies participants into categories such as normal, mild, or moderate to severe based on the total score. However, due to its cultural adaptation, precise cutoff scores and scoring guidelines are best referenced from the validation studies or directly obtained from AKU.

Following the screening by AKUADS to label anxiety and depression separately, the HADS was used. This 14-item self-administered tool is divided into two subscales, each containing seven items, to measure anxiety and depression [17]. Each item is rated on a four-point Likert scale from 0 to 3, allowing participants to rate the frequency of their symptoms [17]. The total score determines the presence of anxiety and depression: a score of 0-7 indicates no anxiety/depression, while a score of 8 and above suggests anxiety/depression.

Both the controls and the patients provided written informed permission. Parental consent was obtained for participants under the age of 18 to ensure ethical compliance and protect the rights of minors involved in the study. This process ensured that parents or guardians were fully informed about the nature of the research, the potential risks, and the confidentiality of participants' data, in accordance with ethical guidelines for research involving minors. Participants received reassurances that their information would be kept confidential. In compliance with the Declaration of Helsinki, this study was approved by the Institutional Ethical Review Board of Azra Naheed Medical College in Lahore.

IBM SPSS Statistics for Windows, Version 27.0 (Released 2020; IBM Corp., Armonk, New York, United States) was used to analyze the data. The descriptive stats were displayed as either count and proportion (%) or mean±standard deviation, depending on the nature of the test. Socio-demographic features and degrees of anxiety and depression were compared with the help of the chi-squared test and independent t-test, respectively. The severity of acne was compared with the levels of anxiety and depression with the help of the Kruskal-Wallis test. For statistical significance, a p-value of less than 0.05 was considered.

## Results

The socio-demographic features of the included study members are summarized in Table 1. Out of 450 participants, females made up a majority of the sample (302, 67.1%), while males accounted for 148 (32.9%). The most significant age group was 23-33 years where 192 (42.7%) have acne, and 281 (62.4%) of the participants were unmarried. Most participants (328, 72.9%) had a bachelor's graduation level or higher. Notably, a significant difference was seen in the case of marital status between participants with and without acne, as a higher proportion of acne cases were single compared to controls (174 (69.6%) vs. 107 (53.5%); p<0.001). Other socio-demographic variables did not display significant differences between the two study groups (p>0.05).

**Table 1:** Socio-demographic characteristics of participants with and without acne vulgaris **: level of significance at p<0.05 The chi-squared test was used to determine the p-values

Characteristic	Total n (%) (n=450)	Cases n (%) (n=250)	Controls n (%) (n=200)	P-value
Gender				
Male	148 (32.9%)	82 (32.8%)	66 (33%)	0.983
Female	302 (67.1%)	168 (67.2%)	134 (67%)	
Age group				
16-22 years	167 (37.1%)	92 (36.8%)	75 (37.5%)	0.987
23-33 years	192 (42.7%)	108 (43.2%)	84 (42%)	
34-40 years	91 (20.2%)	50 (20%)	41 (20.5%)	
Marital status				
Never married	281 (62.4%)	174 (69.6%)	107 (53.5%)	<0.001**
Married	169 (37.6%)	76 (30.4%)	93 (46.5%)	
Educational level				
High school or lower	122 (27.1%)	75 (30%)	47 (23.5%)	0.092
Bachelor's or higher	328 (72.9%)	175 (70%)	153 (76.5%)	

In terms of acne severity, the majority of individuals with acne (154, 61.6%) had moderate acne, followed by mild and severe forms at 44 (17.6%) and 52 (20.8%), respectively (Table 2).

**Table 2:** Severity of acne in the case group according to GAGS GAGS: Global Acne Grading System

Severity level	Percentage (%)	Case group (n=250)
Mild	17.6%	44
Moderate	61.6%	154
Severe	20.8%	52

Table 3 presents the findings related to anxiety and depression scores based on the HADS. The mean anxiety HADS score for the entire sample was 7.18±4.72, with 188 (41.8%) participants classified as anxious. The mean depression HADS score was 9.39±4.10, with 222 (49.3%) participants categorized as depressed. A statistically significant difference was observed in anxiety HADS scores between cases and controls, with acne cases showing higher anxiety levels (17.62±4.85 vs. 6.74±4.40; p=0.027). Depression HADS scores, however, were not significantly different between the two study groups (p=0.643).

**Table 3:** Occurrence of anxiety and depression according to HADS score The HADS score is based on HADS A: the independent t-test was used to get the p-values B: the chi-squared test was used to get the p-values **: level of significance at p<0.05 HADS: Hospital Anxiety and Depression Scale

HADS parameters	Total n (%) (n=450)	Case group n (%) (n=250)	Control group n (%) (n=200)	P-value	χ² (df)	Effect size (Cramér's V)
Score of anxiety (mean±SD)^A^	7.18±4.72	17.62±4.85	6.74±4.40	0.027**	-	-
Anxiety level^B^						
No anxiety at all	262 (58.2%)	128 (51.2%)	134 (67%)	0.076	3.161 (1)	0.084
Anxious	188 (41.8%)	122 (48.8%)	66 (33%)			
Score of depression (mean±SD)^A^	9.39±4.10	9.47±4.20	9.32±4.00	0.643	-	-
Depression level^B^						
No depressed at all	228 (50.7%)	118 (47.2%)	110 (55%)	0.849	0.036 (1)	0.009
Depressed	222 (49.3%)	132 (52.8%)	90 (45%)			

Table 4 scrutinizes the association between socio-demographic factors and anxiety levels among the participants with and without acne. In the acne group, a significantly higher proportion of anxious participants were female (102, 72.9%) compared to males (38, 27.1%; p=0.021), indicating that anxiety was more common among women with acne. Other socio-demographic features, such as age group, marital status, and educational level, did not show statistically significant associations with anxiety (p>0.05).

**Table 4:** Correlation of the level of anxiety with the socio-demographic features among participants (n=450) #: the chi-squared test was used to get the p-values **: level of significance at p<0.05

Factor	Cases		Controls		P-value#	χ² (df)	Effect size (Cramér's V)
	With anxiety n (%) (n=140)	No anxiety n (%) (n=156)	With anxiety n (%) (n=119)	No anxiety n (%) (n=177)			
Gender							
Male	38 (27.1%)	59 (37.8%)	36 (30.3%)	62 (35%)	0.021**	χ² (1)=5.35	0.11
Female	102 (72.9%)	97 (62.2%)	83 (69.7%)	115 (65%)			
Age group							
16-22 years	58 (41.4%)	52 (33.5%)	47 (39.5%)	64 (36.2%)	0.06	χ² (2)=1.71	0.426
23-33 years	54 (38.6%)	73 (46.8%)	50 (42%)	72 (40.7%)			
34-40 years	28 (20%)	31 (19.7%)	22 (18.5%)	41 (23.1%)			
Marital status							
Unmarried	100 (71.4%)	112 (71.8%)	72 (60.5%)	88 (49.7%)	0.02**	χ² (1)=0.21	0.646
Married	40 (28.6%)	44 (28.2%)	47 (39.5%)	89 (50.3%)			
Educational level							
High school or lower	35 (25%)	54 (34.6%)	28 (23.5%)	45 (25.4%)	0.07	χ² (1)=2.37	0.124
Bachelor's degree	105 (75%)	102 (65.4%)	91 (76.5%)	132 (74.6%)			

Among control participants, educational level was significantly linked with depression, with a higher prevalence of depression observed among those with a bachelor's degree in comparison with those having a high school education or lower (123 (82%) vs. 27 (18%); p=0.016). Other socio-demographic factors did not show significant associations with depression in either group (Table 5).

**Table 5:** Correlation of the level of depression with the socio-demographic features among participants (n=450) #: the chi-square test was used to get the p-values **: level of significance at p<0.05

Factor	Case (n=250)		Control (n=200)		P-value#	χ² (df)	Effect size (Cramér's V)
	With depression n (%) (n=152)	No depression n (%) (n=98)	With depression n (%) (n=150)	No depression n (%) (n=50)			
Gender							
Male	50 (32.9%)	40 (40.8%)	55 (36.7%)	20 (40.8%)	0.07	χ² (1)=2.38	0.122
Female	102 (67.1%)	58 (59.2%)	95 (63.3%)	30 (59.2%)			
Age group							
16-22 years	55 (36.2%)	38 (38.4%)	53 (35.3%)	22 (44.3%)	0.05	χ² (2)=1.12	0.561
23-33 years	64 (42.1%)	40 (40.2%)	62 (41.3%)	20 (40.7%)			
34-40 years	33 (21.7%)	20 (20.4%)	35 (23.3%)	8 (16.4%)			
Marital status							
Unmarried	100 (65.8%)	66 (67.3%)	83 (55.3%)	25 (50.2%)	0.03	χ² (1)=0.71	0.391
Married	52 (34.2%)	32 (32.7%)	67 (44.7%)	25 (49.8%)			
Educational level							
High school or lower	40 (26.3%)	30 (30.8%)	27 (18%)	15 (30.4%)	0.016**	χ²(1)=4.86	0.151
Bachelor's degree	112 (73.7%)	68 (69.2%)	123 (82%)	35 (69.6%)			

The severity of acne was not significantly associated with anxiety or depression scores. Median anxiety and depression scores did vary across mild, moderate, and severe acne cases, but these differences did not reach statistical significance (p=0.135 for anxiety and p=0.182 for depression), indicating that acne severity did not significantly influence mental health scores among participants with acne (Table 6).

**Table 6:** Association of acne severity and HADS scores of anxiety and depression among participants with acne (n=250) #: the Kruskal-Wallis test was used to get the p-values The level of significance is at p<0.05 HADS: Hospital Anxiety and Depression Scale

Severity of acne	Acne group	Anxiety median (range)	F-test; p-value#	Depression median (range)	F-test; p-value#
Mild	n=80	7 (4-10)	p=0.135	5 (3-9)	p=0.182
Moderate	n=100	6.5 (4-11)		6 (3-8)	
Severe	n=70	9 (5-13)		7 (4-11)	

## Discussion

This study aimed to examine the association of acne vulgaris with anxiety and depression, in comparison to a control group. Similar to the findings in dermatological research, acne has been associated with elevated levels of anxiety and depression, which may disrupt interpersonal relationships and daily functioning [19]. This study observed that acne prevalence was significantly higher among single participants compared to married participants (69.6% vs. 53.5%; p<0.001). This aligns with Ali et al. [20], who suggested marriage could act as a protective factor against acne. Although the relationship between marital status and acne remains uncertain, some researchers propose that regular sexual activity may positively impact mental and physical health, which could help manage acne [21,22]. In contrast to previous studies where marital status was not significantly linked to acne prevalence [23,24], our results suggest a potential protective association of marriage against acne. However, further exploration of the role of marital status in influencing both acne and mental health outcomes is necessary. Marital status could potentially affect psychological well-being, as individuals in different relationship dynamics may experience varying levels of stress, social support, or self-esteem, which can, in turn, influence both acne severity and mental health. For example, married individuals might experience different levels of emotional support, which could either mitigate or exacerbate the emotional distress related to acne. On the other hand, single individuals, or those going through marital challenges, might experience heightened stress or anxiety that could worsen both their acne and mental health. Exploring these factors in future studies could shed light on the intersection between personal relationships and skin health and help develop more personalized treatments.

The severity of acne varied among cases, with the majority (62.5%) presenting moderate acne, consistent with Kurtalić et al., who reported a similar trend with 80% of acne cases categorized as moderate [25]. This finding underscores that moderate acne is the most common presentation, though it may not necessarily predict psychological severity.

Using the HADS score, we found that anxiety HADS scores were significantly higher in the acne group than in controls (17.62±4.85 vs. 6.74±4.40; p=0.027), consistent with findings by Golchai et al., where anxiety scores were notably elevated among acne patients [26]. In contrast, depression HADS scores did not show a significant difference between groups, mirroring Golchai et al.'s study where depression levels were not significantly higher among acne cases. These findings echo the results of Dalgard et al., who reported a higher prevalence of anxiety disorders in patients with common skin conditions compared to controls (17.2% vs. 11.1%) [27]. However, our results did not indicate a link between acne severity and anxiety or depression, suggesting that psychological impact is not necessarily tied to clinical severity, as Jena and Sahoo also observed [15].

A notable finding in our study was the gender-based difference in anxiety among acne patients, with a higher proportion of anxious females than males (72.9% vs. 27.1%; p=0.021). This gender difference in anxiety aligns with research indicating that female acne patients tend to report greater psychological distress, likely due to the perceived impact on appearance and social interactions [15]. However, similar to Kurtalić et al., our results showed no significant relationship between gender and depression levels, indicating that the impact of acne on depression might be influenced by factors beyond gender [25].

In terms of educational attainment, a significant association was observed only in the control group, where participants with a high school education or lower reported higher levels of depression than those with a bachelor's degree or higher (p=0.016). This is consistent with studies showing that education is protective against depressive symptoms, as higher educational levels are often linked to improved mental health and resilience against stress [28]. The link between lower education and depression in our control group reinforces the idea that educational attainment can be a protective factor against mental health issues [29].

Lastly, our study did not find significant associations between acne severity and HADS scores for anxiety or depression (p=0.135 and p=0.182, respectively). These findings, consistent with Kurtalić et al., suggest that while acne can contribute to psychological distress, the severity of physical symptoms may not directly correlate with the level of psychological impact [25]. This points to the importance of psychological support and mental health screening for all acne patients, regardless of severity, as physical appearance alone does not necessarily predict mental health status.

Regarding the absence of a significant association between depression and acne in this study, it is important to delve deeper into potential contributing factors. One possible explanation could be the sample characteristics of the study, which may not have included individuals with severe or long-standing acne, as the study was limited to a specific age group (16-40 years). The level of depression may vary depending on the chronicity or severity of the acne. For individuals with milder acne, depression may not be as strongly evident, whereas those with more severe or cystic acne might experience more pronounced emotional distress. Another possible explanation could be cultural influences, as the way acne is perceived and its associated psychological effects may differ across cultures. In some societies, there may be a greater stigma associated with mental health or acne, leading individuals to underreport feelings of depression. Additionally, methodological aspects such as the use of a cross-sectional design might not fully capture the long-term impact of acne on depression, as depression might develop or worsen over time with persistent acne. Future studies with a longitudinal design or a more diverse sample might reveal a stronger link between acne and depression, providing more clarity on this relationship.

Our study highlights the significant role of marital status and gender in the psychological impact of acne vulgaris. Acne sufferers, particularly women, were found to be more susceptible to anxiety, emphasizing the need for mental health assessments as part of a comprehensive acne management plan [30]. This aligns with previous research that advocates for a holistic approach to acne treatment, considering both physical and psychological aspects [30]. However, the study has several limitations. The participants were recruited from a specific hospital setting, which may not fully represent the general population, as hospital-based samples often have differing health-seeking behaviors compared to the wider community. While the sample size was considerable, future research could benefit from an even larger cohort and a broader geographic distribution to enhance the generalizability of the findings. Furthermore, other potential confounding factors, such as body image perception and social support, which may influence levels of anxiety and depression, were not assessed in this study. Addressing these variables in future studies could provide a more comprehensive understanding of the psychological impact of acne. Finally, longitudinal studies would be beneficial to observe psychological changes over time in acne patients, which could help further elucidate the relationship between acne severity, mental health, and long-term outcomes.

## Conclusions

This study highlights the psychological impact of acne vulgaris, with acne patients exhibiting significantly higher anxiety levels compared to controls, though depression scores did not differ notably between groups. Marital status appeared to be a protective factor, with single individuals more likely to experience acne than married participants, a finding that aligns with some literature suggesting a positive effect of marital stability on health. Additionally, female acne patients reported higher anxiety levels than males, underscoring the need to consider gender-specific psychological support in acne management. While education correlated with depression levels in controls, it did not show a significant impact within the acne group, suggesting that educational attainment may play a more protective role against depression in general than specifically for acne sufferers. These findings support the integration of mental health evaluations in the treatment of acne vulgaris to address the broader emotional and social challenges that patients may face.
